# Myricetin protects mice against colitis by activating aryl hydrocarbon receptor signaling pathway

**DOI:** 10.29219/fnr.v69.10677

**Published:** 2025-01-22

**Authors:** Tao Xu, Xinyan Qu, Yue Song, Mengxiong Luo, Yuhan Jia, Jia Li, Qingjun Li

**Affiliations:** 1 Shandong University of Traditional Chinese Medicine, Jinan, China; 2 Department of Traditional Chinese Medicine, Taishan District People’s Hospital, Taian, China; 3 Shandong Analysis and Test Center, Qilu University of Technology (Shandong Academy of Sciences), Jinan, China

**Keywords:** myricetin, targeted metabolomics, ulcerative colitis, aryl hydrocarbon receptor, mechanism

## Abstract

**Objective:**

Myricetin is a bioactive compound in many edible plants. We have previously demonstrated that myricetin could significantly protect mice against colitis by regulating Treg/Th17 balance, while underlying mechanism remains unclear. The current study aimed to unravel the potential regulating mechanism of myricetin.

**Methods:**

The concentrations of 22 amino acids in colon were determined using HPLC-MS/MS and principal component analysis (PCA) was performed on the data. MetaboAnalyst was used to detect potential biological pathway influenced by myricetin. The results were further verified using qPCR, molecular docking method, and AhR inhibitor.

**Results:**

Studies had found that the biosynthesis of phenylalanine, tyrosine, and tryptophan; phenylalanine metabolism; and histidine metabolism were the most important pathways related to myricetin. Therefore, the aryl hydrocarbon receptor (AhR), which is closely related to the metabolism of tryptophan, phenylalanine, and tyrosine, was postulated to be the underlying signaling pathways. Furthermore, administration of myricet in significantly increased the relative expressions of CYP1A1 and CYP1B1, whereas AhR inhibitor abolished the amelioration of myricetin on DSS-induced colitis. Moreover, AhR inhibitor weakened the regulatory effect of myricetin on Treg/Th17 balance. Furthermore, the results obtained by the molecular docking method speculated that myricetin could bind to AhR as a ligand and activate AhR.

**Conclusion:**

The results suggested that myricetin could exert its protection against dextran sulfate sodium (DSS)-induced colitis by activating AhR signaling pathway.

## Popular scientific summary

Ulcerative colitis (UC) is a chronic inflammatory disease of the intestine that causes symptoms such as abdominal pain, diarrhea, and weight loss. Currently, drugs for treating UC have side effects or poor efficacy, so finding new treatments is crucial. Myricetin is a natural compound found in many edible plants, such as teas, fruits, and herbs. Studies have shown that myricetin has antioxidant, anti-tumor, and anti-inflammatory effects. In this study, myricetin was found to protect mice from dextran sulfate sodium (DSS)-induced colitis. In addition, metabolomics technology was used to study how myricetin affects amino acid levels in the intestines of mice. The results showed that myricetin changed the concentrations of multiple amino acids, which may be related to myricetin’s anti-inflammatory effect of myricetin. Further studies showed that myricetin may work by activating aryl hydrocarbon receptor (AhR), which is involved in regulating the balance between immune response and environmental factors. This study suggests that myricetin may be a new drug for the treatment of UC and provides new ideas for the development of other herbal therapies.

Ulcerative colitis (UC), causing weight loss, abdominal pain, and bloody diarrhea, is the most common type of inflammatory bowel diseases (IBDs), and the underlying pathophysiology remains unclear. Its incidence and prevalence are rising, which challenge the clinicians and health policy makers (1). Lots of patients take inappropriate doses of medications like aminosalicylates, suffer side effects of glucocorticoids, or receive biological drugs without optimized immunosuppressive therapy (2). So, it is of importance to find new therapeutic strategies against UC.

For thousands of years, plants have been administered to treat a range of pathological conditions, and many natural products within plants have been proven to exert protective effect against UC, which provide an opportunity to develop alternative therapies for UC (3, 4). Many edible plants like teas, fruits, and medicinal herbs contain myricetin, a flavonoid compound with three adjacent hydroxyl groups. Myricetin was documented to possess antioxidative, anticarcinogenic, and anti-inflammatory properties (5–7). Moreover, we have established that myricetin restores the regulatory T cells (Treg) and effector T cells (Th17) balance in mice with colitis induced by DSS (8), whereas the underlying mechanism needs further investigation.

Metabolomics, an efficient tool of omics-based technologies, has become a focus in life science with the rapid development of omics-based approaches. Metabolomics offers a novel way to uncover mechanisms of medicine by determining the changes of metabolites profiles in vivo, and its advantages have been increasingly realized (9). In this study, metabolomics was applied to elucidate the underlying mechanism by which myricetin regulates the Th17/Treg balance. The concentration changes of 22 amino acids were determined, and the potential metabolic pathways were predicted. Subsequently, signaling pathways relating with the potential metabolic pathways were surveyed and verified. This work will provide more information for the study of myricetin and inspire us to find potential bioactive compounds in edible plants, to offer more chances for patients suffering from UC.

## Materials and methods

### Regents and pharmacological compounds

Aryl hydrocarbon receptor (AhR) inhibitor (CH223191) and standards of histidine, phenylalanine, γ-aminobutyric acid, glutamine, lysine, proline, tryptophan, asparagine, aspartic acid, leucine, isoleucine, valine, arginine, threonine, methionine, pyroglutamic acid, serine, glutamic acid, tyrosine, taurine, and alanine were purchased from Sigma-Aldrich. The methanol and acetonitrile for HPLC-MS/MS were supplied by Merck, so was methanol for chromatography. Dextran sulfate sodium (DSS, M.W. = 36–50 kDa) was obtained from MP Bioscience, while myricetin (purity > 95%) was purchased from the National Institutes for Food and Drug Control (Beijing, China).

### Animals and drug administration

The female C57BL/6 mice (17–19 g, 7–8 weeks old) were supplied from the Beijing Vital River Laboratory Animal Technology Co., Ltd. The mice were raised under specific pathogen-free conditions with 12-h light-dark cycles and provided standard laboratory food and water ad libitum. All animal experiments protocols were reviewed and approved by the Animal Ethics Committee of Shandong University of Traditional Chinese Medicine (10-03-2022, SDUTCM20220310551), and all animal experiments were conducted in strict accordance with guidelines of the Shandong Administration Office of Laboratory Animals.

The mice were randomly divided into four groups (*n* = 6), with the following administrations: control group, 2% DSS-only group, myricetin group (gavage, 40 mg/kg), myricetin (gavage, 80 mg/kg). As for the experiment to analyze the role of AhR signaling pathway playing in the protection of myricetin against colitis, the animals were randomly distributed into four groups (*n* = 6), including control group, 2% DSS-only DSS group, myricetin group (gavage, 80 mg/kg), and myricetin (gavage, 80 mg/kg) and CH223191 (10 mg/kg, intraperitoneal injection).

Acute experimental colitis was induced by exposing the mice to 2% DSS for 7 consecutive days. Myricetin, CH223191, or the same volume of vehicle was administered once a day for 1 week behind the induction of colitis. The body weight of mice was recorded every day, and the colon length and disease activity index (DAI) were determined on the day mice euthanized (8).

### Detection of amino acid concentration and data processing

Method described by Song et al. was conducted to detect the concentration of targeted amino acids (10). First, the colonic tissues from different groups were collected and homogenized on ice in 100 μL methanol. Then, the homogenized samples were mixed thoroughly with methanol (300 μL) and water (280 μL), while chloroform was used to exclude other impurities. HPLC-MS/MS (Agilent 6420) was employed to analyze the extracts of colonic samples.

All the raw data were imported into the Agilent Masshunter Quantitative Analysis software to be quantified. Then, the pre-processed data were imported into the SIMCA-P software (version 13.0, Umetrics AB, Sweden) to perform principal component analysis (PCA) (11, 12). Moreover, the SPSS software was used in significance test.

### Metabolomics pathway analysis

One-column compound list of amino acids was submitted into the Pathway Analysis (targeted) module of MetaboAnalyst (*https://www.metaboanalyst.ca*), which is a web-based tool designed to perform comprehensive metabolomic data analysis, visualization, and interpretation, to acquire affected pathways.

### Analysis of myricetin–AhR interaction using the molecular docking method

The interaction between myricetin and the signaling pathway, most associated with affected metabolomics pathways, was analyzed by the molecular docking method. Target protein was searched in the Uniprot database, and its crystal structures were filtered and downloaded. Subsequently, the crystal structures of target protein were opened using Discovery Studio Software, and dehydration and heteroatoms deletion were performed. The primordial ligands and binding sites were selected, and spatial sites were determined by From Current Selection. The structure of myricetin was found in the PubChem database and imported into the Discovery Studio software. Data were obtained after the docking the ligand with the protein by Docks Ligands.

### Quantitative real-time reverse transcription polymerase chain reaction

To analyze whether the investigated signaling pathways are truly affected by myricetin, total RNA of colon samples was extracted using the RNA extraction kit (TaKaRa, 9767), and reverse transcription was performed according to the manufacture’s protocols. Standardized with reference to GAPDH, relative expression levels of genes CYP1A1, CYP1B1, Foxp3, and ROR γt were determined via qRT-PCR subsequently. Sequences of the primers were as follows: GAPDH (forward 5′-CATCACTGCCACCCAGAAGACTG-3′ and reverse 5′-ATGCCAGTGAGCTTCCCGTTCAG-3′); CYP1A1 (forward 5′-CATCACAGACAGCCTCATTGAGC-3′ and reverse 5′-CTCCACGAGATAGCAGTTGTGAC-3′); CYP1B1 (forward 5′-GCCACTATTACGGACATCTT CGG-3′ and 5′-ACAACCTGGTCCAACTCAGCCT-3′); Foxp3 (forward 5′-CCTGGTTGTGAGAAGGTCTT CG-3′ and 5′-TGCTCCAGAGACTGCACCACTT-3′); ROR γt (forward 5′-ACAGCAGGAGCAATGGAAGTC-3′ and 5′-GCAGAGATGATGATGGAAAGCC-3′).

### ELISA determination

Mouse IL-6, IL-22, IL-10, and IL-17 precoated ELISA kits were purchased from Dakewe Bio-Engineering Co., Ltd. (Beijing, China). The concentrations of IL-6, IL-22, IL-10, and IL-17 in the serum and colorectum were determined in accordance with the introductions provided by the manufacturer.

### Antibodies and flow cytometry

Antibodies against mouse CD3 (100216), CD8a (100744), and CD25 (102038) were purchased from BioLegend; Foxp3 (560401), B220 (552094), and CD4 (552775) were purchased from BD Biosciences. Samples of mesenteric lymph nodes (MLN) were smashed through a 70-μm cell filter to prepare single-cell suspensions. In a 96-well plate, 3 * 10^6^ lymphocytes/well were stimulated with PMA, ionomycin, brefeldin, and monensin for 4 h. The proportion of Treg and Th17 cells was determined using flow cytometry.

### Histological analysis

The collected colon samples were fixed in 4% neutral paraformaldehyde and embedded in paraffin. 5-μm colon sections were obtained and stained with hematoxylin and eosin (HE). The histological analysis was done blind by a person who specializes in pathology. Tissue damage was scored as follows: 0) absence of mucosal alterations, 1) presence of lymphoepithelia lesions, 2) damage to the superficial mucosal layer, and 3) severe mucosal damage with lesions reaching deeper tissue layers. Inflammatory cell infiltration was scored as follows: 0) sparse cell infiltration, 1) elevated levels of inflammatory cells infiltration, 2) infiltration that reaches the submucosal layer, and 3) transmural extension of the inflammatory cells infiltration.

### Statistical analysis

Results in this study are expressed as mean ± SD. Differences between groups were analyzed using one-way analysis of variance (ANOVA), and *P* < 0.05 was considered statistically significant.

## Results

### Concentration of amino acids

**Fig. 1:**
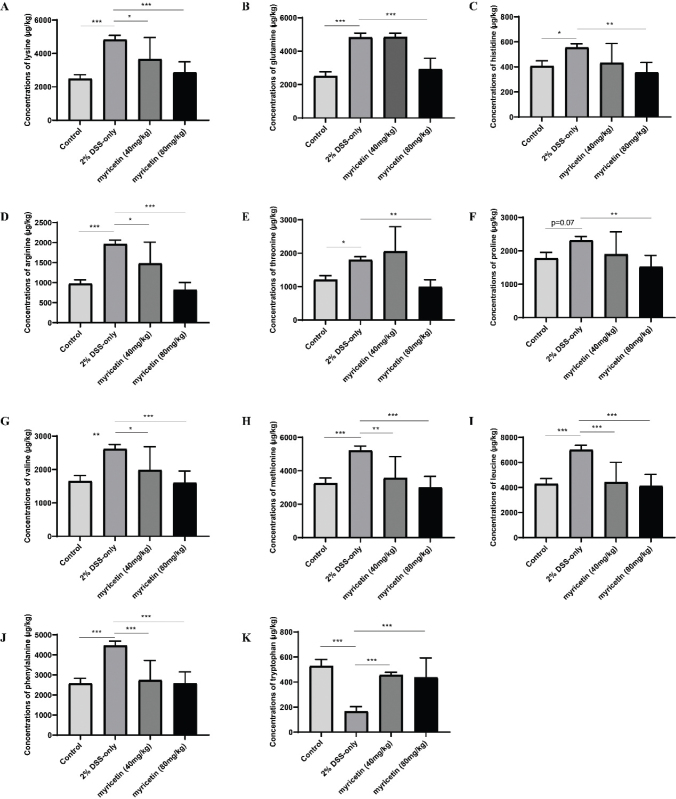
Concentrations of amino acids with significant changes. The concentrations of 22 amino acids were determined by HPLC-MS/MS. Data were pooled from two independent experiments with 6 mice per group.

The concentration changes of 22 amino acids were investigated using HPLC-MS/MS in this study. As shown in Fig. 1, compared with that of mice in normal group, the concentrations of lysine, glutamine, histidine, arginine, threonine, proline, valine, methionine, leucine, and phenylalanine of mice in 2% DSS-only group were significantly increased, whereas tryptophan was significantly decreased. However, myricetin (40 and 80 mg/kg) significantly alleviated the changes of lysine, arginine, valine, methionine, leucine, and phenylalanine, and myricetin (80 mg/kg) significantly alleviated the elevation of glutamine, histidine, threonine, and proline.

### Pattern recognition and biological pathway analysis

**Fig. 2:**
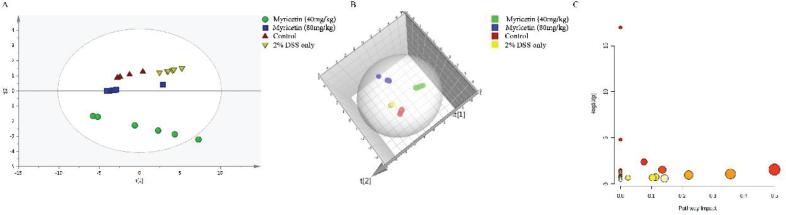
The changes in concentrations of amino acids were analyzed by PCA in the SIMCA-P software (A, B). Green: myricetin (40 mg/kg), Blue: myricetin (80 mg/kg); Red: control; Yellow: 2% DSS only. Impact value of metabolic pathway analysis (C). MetaboAnalyst software was used to find the relevant pathway involved in the myricetin’s alleviation on DSS-induced colitis.

An unsupervised clustering method – PCA – was used to analyze the concentration of amino acids in SIMCA. The PCA score chart (Fig. 2A and B) showed good discrimination between normal control, 2% DSS-only, and myricetin treatment groups. The results indicated that myricetin could induce different types of amino acids changes, and these identified amino acids could be used as biomarkers for the effects of myricetin on DSS-induced colitis.

MetaboAnalyst is a web-based tool to do statistical, functional, and integrative analysis of metabolomics data for users (13). The names of amino acid with significantly changed concentration were imported into MetaboAnalyst to explore potential pathways affected by myricetin. Nine pathways affected by myricetin were shown in Fig. 2C, and more information of these nine pathways was shown in Table 1. The metabolic pathways, including phenylalanine, tyrosine, and tryptophan biosynthesis; phenylalanine metabolism; and histidine metabolism, whose impact was more than 0.2, were selected to be the most important.

**Table 1:** Results from Pathway Analysis

Metabolic Pathways	Total	Hits	Raw p	FDR p	Impact
Phenylalanine, tyrosine and tryptophan biosynthesis	4	1	0.028926	0.45258	0.5
Phenylalanine metabolism	12	1	0.084509	0.70988	0.35714
Histidine metabolism	16	1	0.1112	0.84918	0.22131
Tryptophan metabolism	41	1	0.26262	1	0.14305
Arginine and proline metabolism	38	2	0.029547	0.45258	0.13566
Alanine, aspartate and glutamate metabolism	28	1	0.18709	1	0.11378
Cysteine and methionine metabolism	33	1	0.21693	1	0.10446
Arginine biosynthesis	14	2	0.00421	0.11787	0.07614
Glycine, serine and threonine metabolism	34	1	0.22278	1	0.02408

The Total is the total number of compounds in the pathway; the Hits is the actually matched number from the user uploaded data; the Raw p is the original p value calculated from the enrichment analysis; the FDR p is the p value adjusted using False Discovery Rate; the Impact is the pathway impact value calculated from pathway topology analysis.

### The interaction between myricetin and AhR and the effect of myricetin on the relative expression levels of CYP1A1 and CYP1B1

**Fig. 3:**
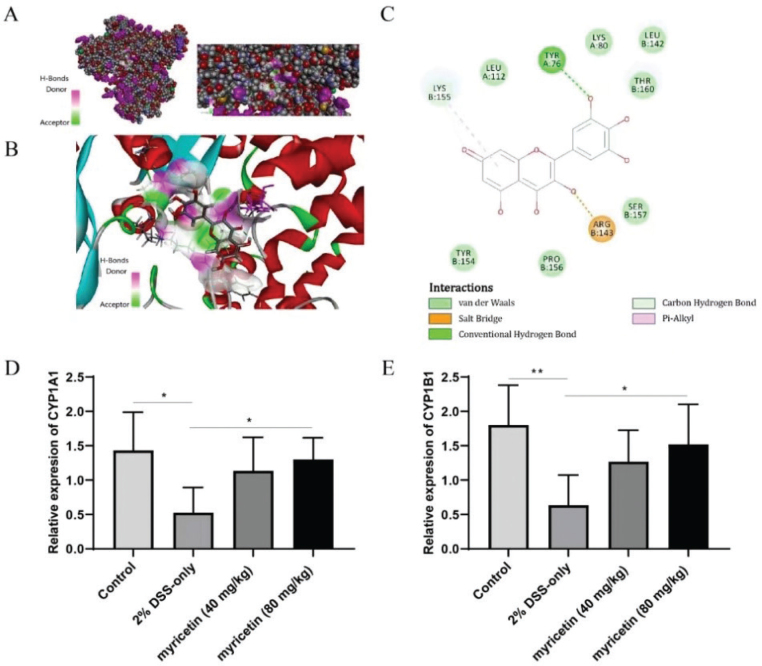
Global and local overview of the best binding model of AhR with myricetin. CPK shape representing is AhR. Stick shape representing is myricetin. Local overview of the best binding model of AhR with myricetin (A). CPK shape representing is AhR. Stick shape representing is myricetin (B). AhR–myricetin complex after docking and molecular dynamic simulation. The colors of the spheres and pointed lines indicate the interaction type between myricetin and residues side chain from AhR (C). qRT-PCR was used to detect the relative expression levels of CYP1A1 and CYP1B1 (D–E). Data were pooled from two independent experiments with 6 mice per group.

It was suggested that myricetin could potentially activate AhR. According to previous publishes, AhR is closely related with the metabolism of tryptophan, phenylalanine, and tyrosine, especially tryptophan. Many of the metabolites of tryptophan, including indole-3-acetic acid (IAA) and indolepopionic acid (IPA), are ligands of AhR and play important roles in regulating immune homeostasis (14, 15). Therefore, AhR was postulated to be the underlying signaling pathways by which myricetin exerting its function. Flavonoids, such as baicalein, quercetin, and galangin, obtained from dietary materials have been reported as AhR ligands. Therefore, it was reasonable to further analyze the interaction between myricetin and AhR. The myricetin–AhR interaction was analyzed by the molecular docking method. As shown in Fig. 3A–C, the results indicated that myricetin could bind to the catalytic site of AhR to form a stable AhR-Myricetin complex. The CDOCKER_ENERGY and CDOCKER_INTERACTION_ENERGY values of Myricetin for AhR were, respectively, –35.3111 and –44.1125. To further verify the effect of myricetin on the AhR signaling pathway, the expression levels of downstream effector genes of AhR including CYP1A1 and CYP1B1 were determined by Quantitative Real-time Reverse Transcription Polymerase Chain Reaction (qRT-PCR). It was shown in Fig. 3D–E, that administration with myricetin could significantly increase the relative expression levels of CYP1A1 and CYP1B1.

### CH223191 prevented the amelioration of myricetin on DSS-induced colitis

**Fig. 4:**
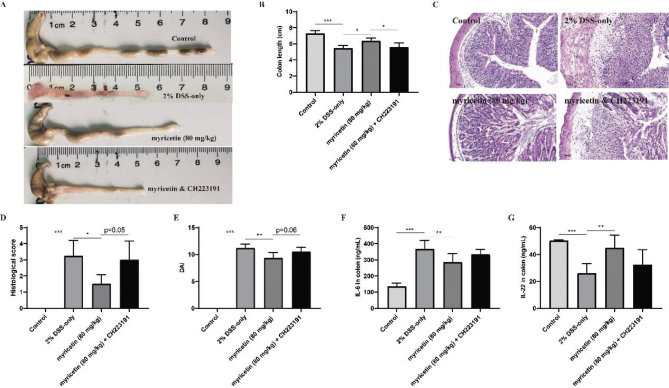
Effect of myricetin on DSS-induced colitis; meanwhile, CH223191 was used to inhibit AhR signaling. Pictures of colon (A), colon length (B), histopathological changes of colon (C), histological score (D), and DAI (E) were determined at the end of the experiment. The expression levels of IL-6 and IL-22 in colonic tissues were detected using ELISA (F and G) (*n* = 6).

Furthermore, CH223191 (AhR inhibitor) was used to determine the underlying protective mechanism of myricetin on DSS-induced colitis. The colon length, histological score, and DAI were detected, and it was found that there was no difference between the myricetin + CH223191 group and the DSS-only group, while it was significantly different between the myricetin (80 mg/kg) group and the myricetin + CH223191 group (Fig. 4A–E). In addition, the expression levels of IL-6 and IL-22 were determined. As shown in Fig. 6F, myricetin significantly decreased the pro-inflammatory cytokine IL-6, and CH223191 prevented the effect of myricetin. IL-22, which is the downstream protein of AhR, was significantly upregulated by myricetin. These results together suggested that myricetin exhibited its protective effect by activating AhR.

### CH223191 impaired the regulation of myricetin on Treg/Th17 balance

**Fig. 5:**
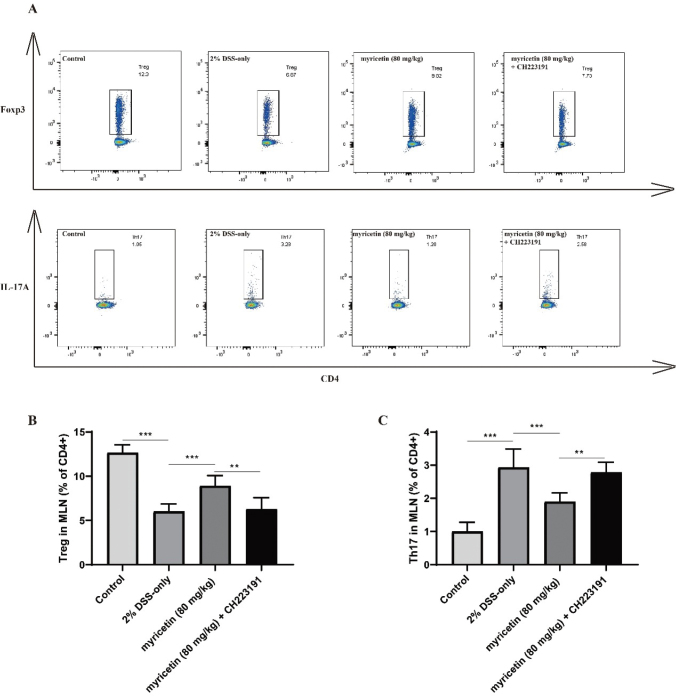
Effect of myricetin on Treg and Th17 cells from MLN. MLN cells were isolated from four mice groups, stained for Treg and Th17 cells, and analyzed by flow cytometry. Representative data were shown in Figure A, indicating that the proportion of Treg and Th17 cells was depicted in (B) and (C) (*n* = 6).

**Fig. 6:**
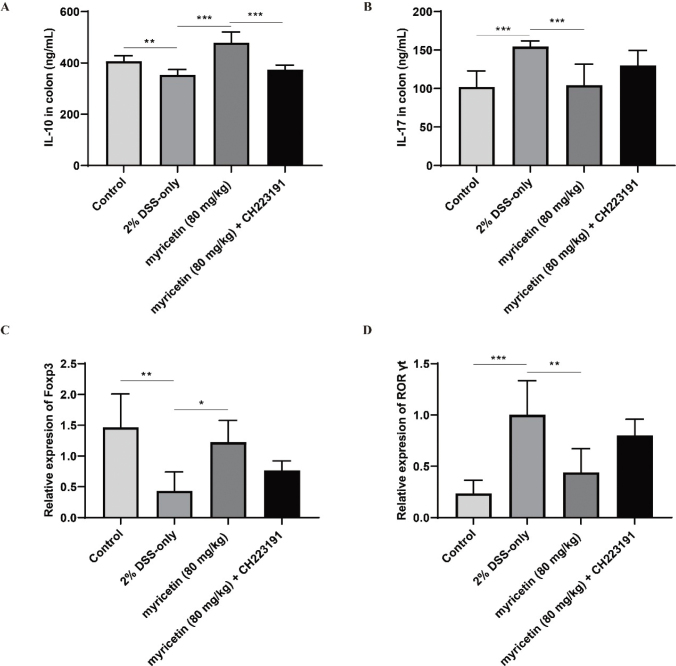
Effect of myricetin administration on IL-10, IL-17, Foxp3, and ROR γt. ELISA was used to determine the levels of IL-10 and IL-17 (A–B), while qRT-PCR was used to detect the relative expression levels of Foxp3 and ROR γt (C–D). Data were pooled from two independent experiments with 6 mice per group.

It has been found that myricetin administration helps to restore the immune balance by regulating Treg and Th17. In this study, the proportions of Treg and Th17 in MLN were determined using flow cytometry. As shown in Fig. 5B–C, myricetin significantly increased the proportion of Treg cells in MLN, while it significantly decreased the proportion of Th17 cells in MLN. In addition, myricetin significantly promoted the secretion of IL-10 and reduced the level of IL-17 (Fig. 6A–B), which is produced by Treg cell and Th17 cell, respectively. Moreover, the transcription factors of Treg and Th17 cells (Foxp3 and ROR γt) were detected by qRT-PCR. As shown in Fig. 6C–D, the relative expression level of Foxp3 was significantly downregulated, and ROR γt was significantly upregulated, compared to mice in the control group. Following myricetin administration, this tread was improved, while CH223191 significantly impaired the effect of myricetin.

## Discussion

Amino acids are compounds required by intestinal mucosal cells to synthesize active proteins with signaling function (16). Some studies have demonstrated that amino acid levels in serum are different between colitis patients and healthy people, suggesting a relationship among amino acid profiles and colitis (17). In the current study, it was found that myricetin could improve the significant changes of the concentrations of tryptophan, lysine, proline, valine, histidine, phenylalanine, glutamine, arginine, threonine, methionine, and leucine of mice caused by intestinal inflammation. It suggested that the underlying mechanism by which myricetin alleviated colitis could be investigated by taking advantage of metabolomics. Finally, nine pathways affected by myricetin were found, while phenylalanine, tyrosine, and tryptophan biosynthesis; phenylalanine metabolism; and histidine metabolism were selected to be the most important.

It has been found that histidine, tryptophan, and phenylalanine are strongly predictive to discriminate intestinal bowel disease patients and healthy controls (18). Phenylalanine, tyrosine, and tryptophan have been demonstrated to be the potential biomarkers for gastro-esophageal cancer (19). Histidine is one kind of conditionally essential amino acids, and it can effectively scavenge the hydroxyl radical and singlet oxygen (20, 21). The role of histidine metabolism in the pathophysiology of IBD and the therapeutic strategy for IBD has been demonstrated (22–24).

In addition, phenylalanine, tyrosine, tryptophan, and histidine play important roles in the AhR signaling pathway, especially tryptophan (25–30). Phenylalanine, tyrosine, and tryptophan are three kinds of aromatic amino acids, phenylalanine is the precursor of non-essential amino acid tyrosine, and tryptophan is required to produce serotonin, kynurenine, tryptamine, and indol-3-acetaldehyde, IAA and IPA. Some of these metabolites including IAA and IPA are ligands of AhR, a ligand-dependent cytoplasmic transcription factor, which is a member of Per-ARNT-Sim-basic helix-loop-helix protein family (31). It was first identified as a mediator of dioxins, and increasing evidence shows that AhR is closely related with the intestinal inflammation (32–34). It was found that AhR could ameliorate inflammation induced by colitis by regulating the balance of Tregs and Th17 cells (35). As a result, AhR signaling pathway was suggested to be the underlying mechanism by which myricetin could exhibit the protection against colitis.

Then, the interaction between myricetin and AhR was analyzed by the molecular docking method. The result suggested that it could bind to the catalytic site of AhR to activate AhR. Furthermore, the relative expression levels of CYP1A1 and CYP1B1, which were induced by AhR activation, were determined to verify the speculation. It was found that myricetin could promote the transcription of CYP1A1 and CYP1B1. In addition, CH223191 was used to further elucidate the role of AhR in the remission of colitis by myricetin. It was found that the alleviation of myricetin was reduced by CH223191.

The abnormal immune function is closely related to UC, and the immune imbalance of CD4^+^ T cells plays a key role in the pathogenesis of UC. Treg and Th17 cells are important subsets of CD4^+^ T cell, and their coordination and balance is pivotal in maintaining the immune balance. Many studies have shown that the imbalance of Treg/Th17 may be the most direct and important factor in the pathogenesis of UC. It has been found previously that myricetin regulates the balance of Treg/Th17 to protect mice with colitis. In this study, it was demonstrated that the regulation of myricetin on the Treg/Th17 balance was impaired by CH223191, the inhibitor of AhR signaling pathway.

These results taken together suggested that myricetin restored Th17/Treg balance to protect mice against DSS-induced colitis by activating AhR signaling pathway, which is closely related to immune balance, intestinal inflammation, and environment (36, 37).
